# A Case Report on Congenital Cytomegalovirus

**DOI:** 10.7759/cureus.42792

**Published:** 2023-08-01

**Authors:** Arley K Rodriguez, Lindsay Tjiattas-Saleski

**Affiliations:** 1 Obstetrics and Gynecology, Edward Via College of Osteopathic Medicine-Carolinas, Spartanburg, USA; 2 Emergency Medicine, Edward Via College of Osteopathic Medicine-Carolinas, Spartanburg, USA

**Keywords:** congenital infection screening, petechiae, thrombocytopenia, valgancyclovir, cytomegalovirus (cmv), congenital infection

## Abstract

Cytomegalovirus (CMV) is the most common congenital infection worldwide and in the United States. The majority of healthy adults who acquire CMV infections have few symptoms and no long-term consequences, though this is not the case for certain groups, including neonates infected in utero. This infection can lead to permanent sequelae, including death. Despite this, congenital cytomegalovirus (cCMV) is not well known among women of childbearing age. Women are more informed about neural tube defects, fetal alcohol syndrome, Down syndrome, and toxoplasmosis than they are about cCMV, although these pose less threat to the newborn. This is a case of a newborn presenting with petechiae, thrombocytopenia, and direct hyperbilirubinemia due to cCMV infection. The initial diagnosis was congenital sepsis, not cCMV. This case report highlights the importance of including a TORCH (toxoplasmosis, others, such as syphilis, rubella, CMV, and herpes) panel when considering abnormal neonatal findings. Diagnosing cCMV is critical, especially because untreated infection can cause permanent sequelae, including death.

## Introduction

Cytomegalovirus (CMV) is a herpesvirus (herpesvirus type 5) that is shed in urine, saliva, cervical mucus, semen, stool, and breast milk. Over half of the adults have been infected with CMV by age 40 [1]. A majority of healthy adults who acquire CMV infections have few symptoms and no long-term consequences. Once a person becomes infected, the virus remains latent and may reactivate occasionally. CMV infection can result in serious health problems in certain groups, including infants infected in utero (congenital cytomegalovirus, or cCMV), premature infants, and persons with compromised immune systems (e.g., HIV, transplant surgery). CMV is included in the list of infections that can be passed via the placenta to the baby during pregnancy, commonly referred to as "TORCH" (toxoplasmosis, others such as syphilis, rubella, CMV, and herpes). Though cCMV is the most common congenital infection worldwide, many women are not educated about it [1-4]. The exact reasoning behind this is not known. This case report aims to change this narrative in hopes of increasing awareness of cCMV to both women and providers worldwide. The outcome of congenital cytomegalovirus depends upon the clinical pattern of disease at birth and the antiviral treatment provided in the newborn period. It is postulated that underdiagnosis of cCMV is largely due to approximately 90% of neonates being asymptomatic upon presentation. Pregnant women can decrease their chances of being infected by CMV by reducing contact with saliva and urine from babies and young children [5]. Proper hygienic measures are the best prevention for this infection [5]. Early detection of cCMV is crucial because this infection can cause detrimental, and often permanent, consequences.

## Case presentation

A 6 lb 7.6 oz (2937 g) early-term 37-week gestational age newborn female was vaginally and spontaneously born to a Group B *Streptococcus *(GBS)-negative and blood type O negative mother. The mother was HIV-, hepatitis B-, and rapid plasma reagin (RPR)-nonreactive. She had a history of herpes simplex virus 1 (HSV-1) but did not report lesions or outbreaks at delivery. The pregnancy was complicated by suboptimal prenatal care, severe maternal depression requiring hospitalization, maternal chlamydia infection in the first trimester treated with an adequate course of azithromycin, maternal anemia, and maternal urine drug screen positive for marijuana. The mother failed a one-hour glucola and did not follow up for a three-hour glucola.

**Figure 1:**
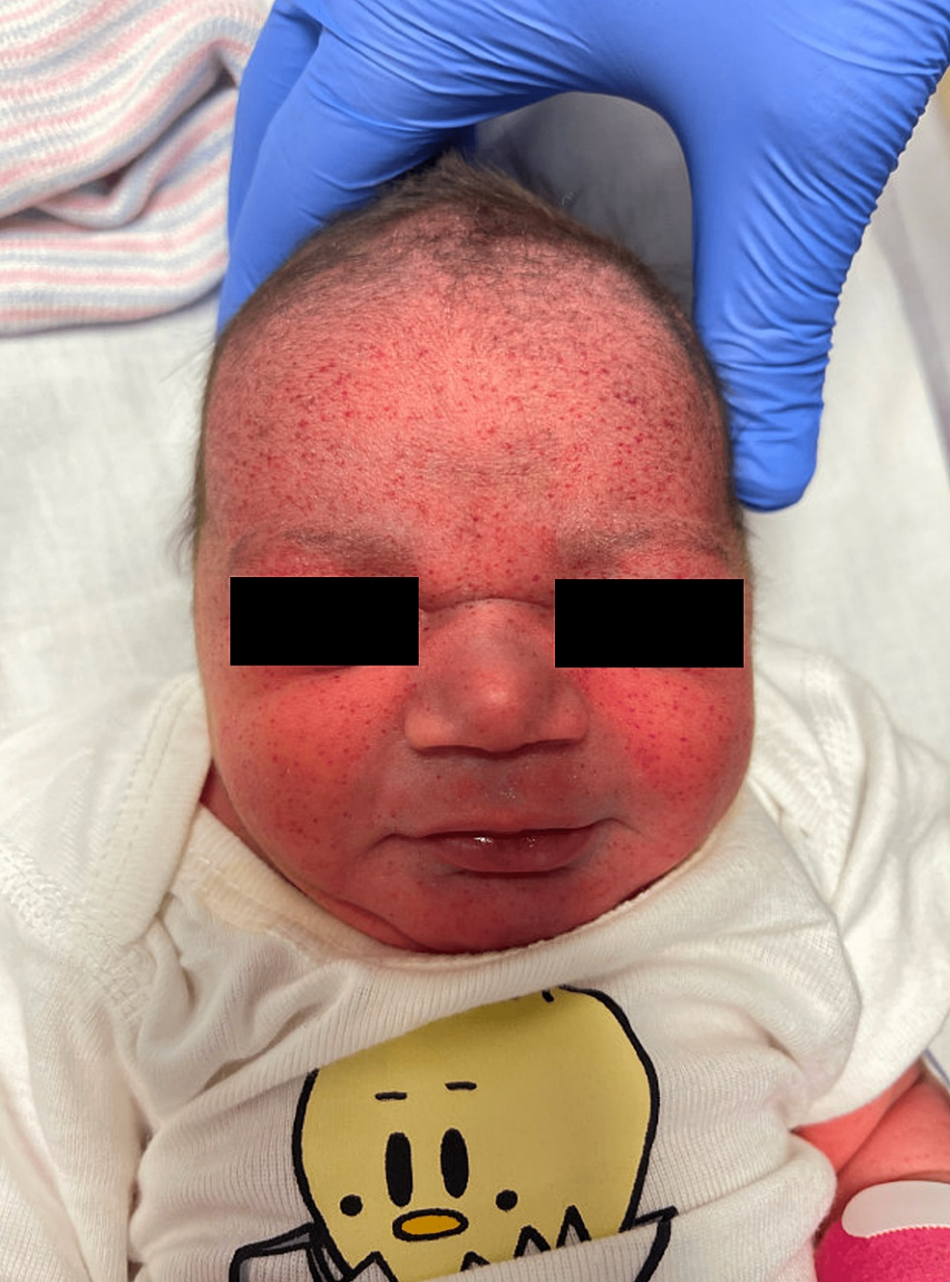
Petechial rash on the face of the patient

**Figure 2:**
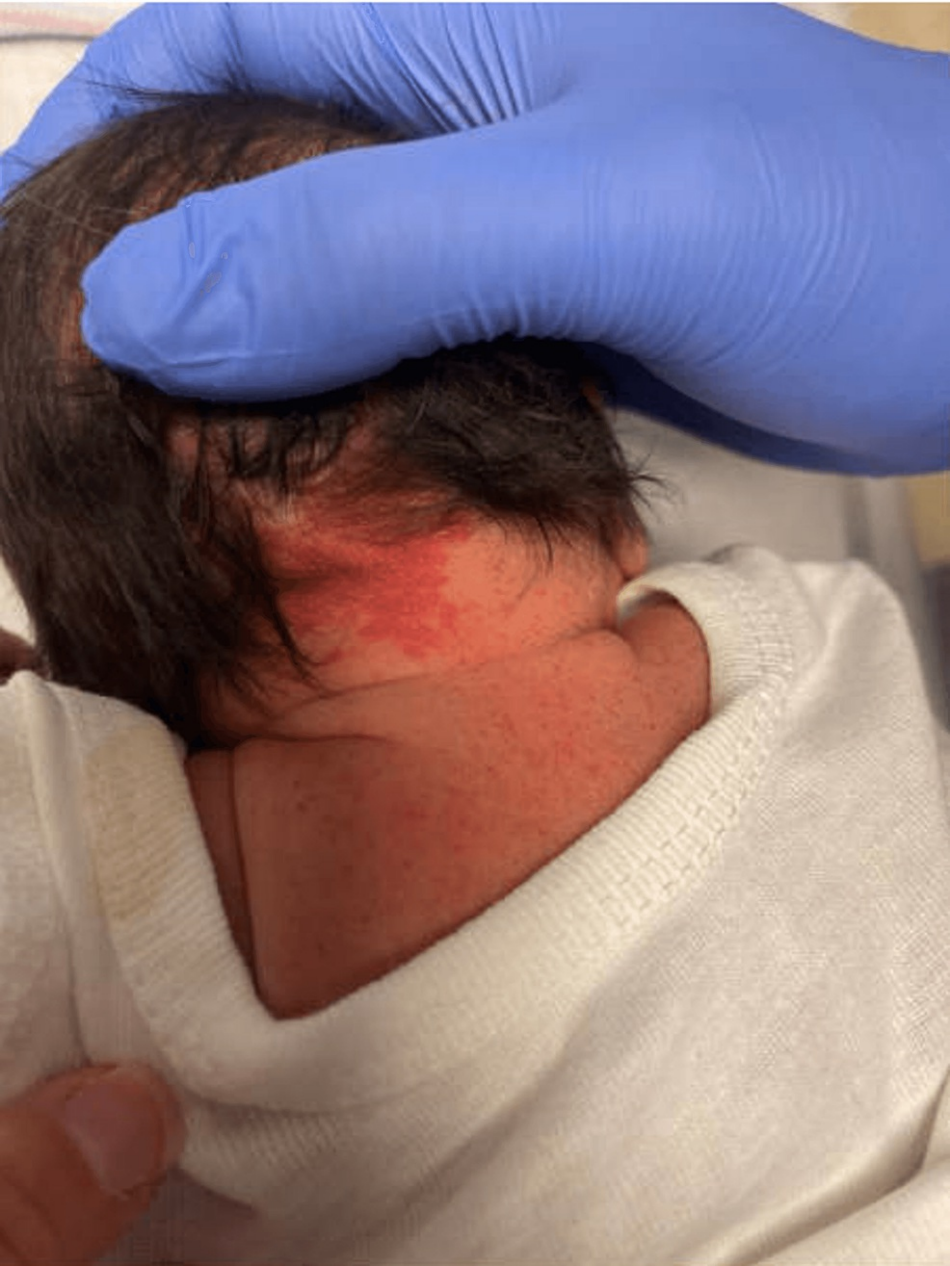
Petechial rash on neck and back

Apgar scores were 7 and 8 at one and five minutes, respectively. The patient was noted to have diffuse petechial rash most prominent on the forehead and face but extending to the trunk, abdomen, and bilateral upper and lower extremities (Figures 1, 2). There was no palpable hepatomegaly or splenomegaly. A small head circumference was measured at 31.1 cm (normal, 35 cm).

Due to a concern for possible congenital viral infection or sepsis, the patient was transferred and admitted to the NICU for close monitoring. A sepsis screen including blood cultures, complete metabolic panel (CMP), complete blood count (CBC), arterial blood gas (ABG), and lactic acid was performed. The patient was started on empiric antibiotics, pending blood cultures. Additionally, a TORCH infection panel was collected. An ultrasound echoencephalogram was ordered to evaluate the small head circumference.

The patient’s laboratory results revealed thrombocytopenia (53K; normal range: 150K-450K) and elevated direct hyperbilirubinemia (1.4 mg/dL; normal range: 0-0.2 mg/dL). Urine polymerase chain reaction (PCR) was positive for CMV. Blood and urine cultures remained negative; thus, antibiotics were discontinued three days after admission. The patient’s urine drug screen was positive for marijuana. Ultrasound echoencephalogram showed a normocephalic head and no evidence of intracranial or periventricular calcifications. Head circumference was noted to be within the 13th percentile after remeasurement.

The patient received phototherapy upon admission due to elevated direct bilirubin levels; phototherapy was discontinued once levels stabilized. The patient received intravenous (IV) ganciclovir 6 mg/kg every 12 hours for 10 days. The patient’s platelet count increased from 53K to 74K (normal range: 150K-450K); therefore, the patient was transitioned to oral valganciclovir 16 mg/kg every 12 hours. Outpatient follow-up with pediatric infectious disease two days after discharge was scheduled, with plans to assess the patient’s hearing and vision at this visit. The patient’s mother was directed to follow up with a primary care physician after concluding the appointment with pediatric infectious disease.

## Discussion

Diagnosis and clinical signs

Despite its health, social, and economic burdens, cCMV infection often goes undetected at birth because screening programs, both in pregnant women and newborns, have not been well-developed or routinized. Primary maternal CMV infection may be suspected if a woman has symptoms consistent with adult infection, including fever, sore throat, fatigue, and swollen glands [6-8]. Viral DNA can be detected by PCR. Maternal viremia in women with proven primary CMV is associated with fetal infection; fetal diagnosis relies on amniocentesis [8], although cCMV can be suspected during prenatal ultrasound. It is very common for pregnant women to have a routine ultrasound at 18-20 weeks to evaluate fetal well-being. Ultrasound findings that are sensitive, but not specific, for cCMV include microcephaly, periventricular calcifications, ventricular dilatation, and pseudocysts [8]. Following delivery, proof of cCMV infection requires virologic detection of CMV in urine, oral fluids, respiratory tract secretions, blood, or cerebrospinal fluid obtained within two to four weeks [9]. Signs and symptoms of congenital cytomegalovirus include petechiae in 76%, jaundice in 67%, hepatosplenomegaly in 60%, microcephaly in 53%, intrauterine growth retardation in 50%, chorioretinitis/optic atrophy in 20%, purpura in 13%, and seizures in 7% of patients [10-12]. Laboratory findings suggestive of infection include elevated AST in 83%, conjugated hyperbilirubinemia in 81%, thrombocytopenia in 77%, and elevated cerebrospinal protein in 46% of patients [10-12]. Radiographic findings in the brain are abnormal in approximately 50%-70% of children with symptomatic infection at birth, with the most common finding being intraventricular calcification in 79% of patients [10-12].

Treatment

Intravenous (IV) ganciclovir and its orally available prodrug, valganciclovir, are the first-line antiviral agents of choice for treating cCMV infection [13]. The dose of ganciclovir is 6 mg/kg administered IV every 12 hours [13]. The dose must be adjusted in neonates with renal failure. Infants may be transitioned to oral valganciclovir 16 mg/kg every 12 hours if they are clinically stable and able to take oral medications [13]. The antivirals, foscarnet and cidofovir, are reserved for cases of refractory CMV infection, ganciclovir resistance, ganciclovir toxicity, and coinfection with adenovirus [13]. Neonates with symptomatic cCMV disease with or without central nervous system involvement have improved audiologic and neurodevelopmental outcomes at two years of age when treated with oral valganciclovir for six months [13]. Significant neutropenia occurs in one-fifth of neonates treated with oral valganciclovir and in two-thirds of neonates treated with parenteral ganciclovir [13]. The American Academy of Pediatrics recommends that absolute neutrophil counts should be performed weekly for six weeks, then at eight weeks, then monthly for the duration of antiviral treatment, and serum aminotransferase concentration should be measured monthly [13]. In the 10% of cCMV infections that are symptomatic, treatment with antiviral medications in early infancy appears to reduce long-term sequelae; however, further longitudinal studies of treated infants are needed to fully understand the impact of antiviral treatment on long-term disability [14,15]. Hearing evaluations are recommended every three to six months during the first three years of life and annually thereafter until at least 18 years of age [15-17]. Central nervous system manifestations (intellectual disability, cerebral palsy, and seizures) may require special education services [16,17]. Quantification of viremia may be helpful in predicting long-term outcomes, with a number of studies identifying an association between viral load and hearing loss [16,17].

## Conclusions

CMV infection continues to be the most common congenital infection, with profound health, social, and economic burdens. As most affected infants are asymptomatic or present with nonspecific symptoms, timely diagnoses can be a challenge. It is not uncommon for neonates to present after birth with a nonspecific rash, as was seen with this patient, so it is important to perform further workup. This case report highlights the importance of including a TORCH workup when considering abnormal neonatal findings. Diagnosing cCMV is crucial because untreated infection can cause permanent sequelae, including death. A clinical practice guideline and standardized screening for cCMV could result in increased timely diagnosis, decreasing detrimental sequelae.
